# Resilience in aid workers in disaster and emergencies: a systematic review and thematic analysis

**DOI:** 10.3389/fpsyg.2025.1540892

**Published:** 2025-06-18

**Authors:** Mahdi Sadeghi, Zohreh Ghomian, Abbas Ebadi, Sakineh Rakhshanderou, Pirhossein Kolivand

**Affiliations:** ^1^Department of Health in Disasters and Emergencies, School of Public Health and Safety, Shahid Beheshti University of Medical Sciences, Tehran, Iran; ^2^Safety Promotion and Injury Prevention Research Center, Institute for Health Sciences and Environment, Shahid Beheshti University of Medical Sciences, Tehran, Iran; ^3^Nursing Care Research Center, Clinical Sciences Institute, Baqiyatallah University of Medical Sciences, Tehran, Iran; ^4^Department of Public Health, Environmental and Occupational Hazards Control Research Center, School of Public Health and Safety, Shahid Beheshti University of Medical Sciences, Tehran, Iran; ^5^Department of Health Economics, School of Medicine, Shahed University, Tehran, Iran

**Keywords:** resilience, aid workers, rescuers, crisis, disaster, emergencies

## Abstract

**Introduction:**

Resilience is a critical attribute for aid workers operating in disasters and emergencies, as it directly influences their ability to cope with high-stress environments’ psychological and physical challenges. Understanding the components of resilience can inform targeted interventions and training programs, ultimately fostering a more robust and adaptable workforce capable of meeting disaster response demands.

**Objectives:**

This study aimed to identify components of resilience in disaster and emergency aid workers by systematic review and thematic analysis.

**Methodology:**

The systematic review adhered to the PRISMA protocol, searching various databases for resilience studies related to disaster aid workers from 1989 to November 2023. The study’s protocol is registered in PROSPERO under the registration number CRD42024508783. Articles were obtained from data resources such as Scopus, PubMed, Web of Science, and Google Scholar. At first, the keywords were determined based on the title and topic of the research, MeSH, previous texts, and the opinions of researchers and experts, and the search strategy was determined based on the keywords for each database. Inductive content analysis was used to identify components of resilience.

**Results:**

From 3,198 searched studies, 17 were included in the final analysis. We identified five key components of resilience among disaster and emergency aid workers, which were categorized into two main groups: traits and process. Resilience traits include (1) health status, (2) essence and personality, (3) capability and competency, and resilience process includes (1) support platforms, and (2) organization and job.

**Conclusion:**

The study’s results can greatly help to understand the concept of resilience in disaster and emergency aid workers, which will ultimately serve as a guide for officials and researchers in planning and appropriate interventions to promote resilience in aid workers.

**Systematic review registration:**

The study’s protocol is registered in PROSPERO under the registration number CRD42024508783. https://www.crd.york.ac.uk/PROSPERO/view/CRD42024508783.

## Introduction

Resilience, often broadly defined as the ability to positively adapt and thrive in the face of adversity, is increasingly recognized as a critical attribute for disaster and emergency aid workers (Fletcher and Sarkar, 2013). Given the context-specificity of resilience, this review operationally defines it as the sustained capacity of aid workers to maintain well-being and professional efficacy under disaster-related stressors. While some conceptualize resilience as an inherent personality trait, others view it as a dynamic process involving interactions between the individual and their environment (Schreiber et al., 2019).

Resilience has been studied across disciplines, from psychology to organizational leadership, and significant consensus has emerged regarding its multidimensional nature. Notably, early foundational work by Masten (2001) framed resilience as “ordinary magic,” emphasizing its basis in adaptive systems rather than extraordinary traits. Ledesma (2014) synthesizes resilience theories into three key frameworks: trait-based, process-oriented, and outcome-focused models (Masten, 2001). These frameworks highlight the interplay between individual characteristics (e.g., optimism, self-efficacy) and environmental factors (e.g., social support, organizational resources) in shaping resilience (Ledesma, 2014). On the other hand, resilience is not a monolithic construct; its expression and manifestation are deeply intertwined with the socio-cultural context in which it is observed (Cutter, 2016; Xie and Wong, 2021; Bundhoo, 2018). A universally applicable definition of resilience may fall short of capturing the nuances of how individuals and communities navigate adversity in specific settings (Buckingham and Brodsky, 2021; Kaplan, 2002). Therefore, to achieve a comprehensive understanding of resilience, it is crucial to examine its constituent elements and characteristics within a defined community, considering the unique challenges, resources, and cultural values that shape its trajectory (Kirmayer et al., 2009; Bonanno et al., 2015). Resilience stems from the complex interaction of individual and environmental factors, which necessitates the use of multi-systemic models for comprehensive understanding (DeLuca et al., 2022; Schäfer et al., 2024). Mouton (2022) demonstrates that enhancing psychological components improves aid worker effectiveness and mental health in high-stress disaster settings (Schäfer et al., 2024). Given resilience’s crucial role in disaster response, promoting resilience-building programs alongside careful selection is essential (Mouton, 2022). However, research in this area remains limited, particularly regarding the long-term impact of interventions and cultural factors (Forbes and Fikretoglu, 2018; Brooks et al., 2015). Expanding on this, psychological interventions like exposure therapy and mindfulness have shown effectiveness in reducing PTSD among first responders (Guo et al., 2022). Therefore, a holistic approach, considering both individual and environmental factors, is vital for understanding and fostering resilience.

Providing healthcare services during crises and disasters places significant strain on personnel, exposing them to risks that can lead to both physical and psychological health issues (Khankeh et al., 2022). Studies have shown that rescuers are at higher risk of developing chronic diseases such as hypertension, cardiovascular disease, and digestive problems due to job stress and demanding work conditions (Bonanno et al., 2011). Research indicates that the adverse effects of such experiences may be long-lasting. Longitudinal studies of 9/11 rescue workers reveal the enduring psychological toll of trauma, with 9.7% experiencing active post-traumatic stress disorder (PTSD) more than a decade later, alongside 7.9% recovered and 5.9% partial PTSD cases (Bromet et al., 2016). In the United States, PTSD prevalence is substantially higher among rescue personnel compared to the general population, affecting approximately one in three rescuers versus one in five individuals in the general population (Mao et al., 2018). Among high-risk groups such as first responders, this rate increases significantly, with PTSD prevalence estimated at 18% in first responders and 16% in emergency physicians (DeLucia et al., 2019). Additionally, among first responders to The World Trade Center (WTC) Disaster, common mental disorders including depression, anxiety, substance abuse, and sleep disorders were reported. These disorders were often correlated with PTSD (Pietrzak et al., 2014). The study by Kilpatrick et al. (2013) demonstrated a positive correlation between exposure to traumatic events and the prevalence of PTSD. The significant impact of high-stress work and trauma exposure on these populations, as evidenced by studies (Violanti et al., 2019; Harvey et al., 2016), is highly relevant to the current context (Mesa-Vieira et al., 2024; Choi et al., 2024; Testoff et al., 2016). However, PTSD prevalence varies due to examination methods (Kessler et al., 2005) and pandemic conditions (Ghahramani et al., 2023), necessitating careful consideration of methodological and temporal factors (Dückers et al., 2016; Koenen et al., 2017).

Given the widespread nature of accidents and disasters, relief organizations cannot effectively fulfil their missions without a sufficient and capable workforce (Demiroz and Haase, 2020). On the other hand, Promoting first responders’ mental and physical health is essential for their ability to respond to incidents. One effective strategy in this regard is to promote resilience (Pink et al., 2021). The increasing importance of this concept has led to resilience in disaster management gaining increasing attention in recent decades (Demiroz and Haase, 2020). Resilience has become central to disaster risk management and has evolved significantly recently. This growing focus has led to diverse interpretations and approaches to resilience within the field (Barati, 2009). Resilience can significantly improve performance by reducing stress in the workplace (Hartmann et al., 2020), and highly resilient healthcare aid workers experience fewer negative psychological effects and demonstrate greater effectiveness in their work (Mao et al., 2019).

The study of resilience in disaster aid workers is a vital and evolving research domain with significant implications for occupational health and disaster response effectiveness. While resilience plays a critical role in mitigating psychological distress, its practical application must account for systemic and cultural barriers. A comprehensive approach, incorporating resilience alongside broader psychological interventions and structural improvements, is necessary to support first responders effectively. Finally, expanding context-specific research will enhance the applicability of resilience frameworks across diverse settings, ensuring more effective and culturally relevant interventions. On the other hand, this knowledge can help develop appropriate assessment tools to evaluate their well-being and resilience effectively. This systematic review aims to address existing gaps by identifying the key components of resilience in disaster and emergency aid workers, providing a foundation for the development of targeted interventions and develop appropriate assessment tool.

## Methodology

This systematic review, pre-registered in PROSPERO (CRD42024508783) and adhering to PRISMA (Preferred Reporting Items for Systematic Reviews and Meta-Analyses) guidelines (Page et al., 2021) employed a multi-faceted approach to data extraction and synthesis due to the heterogeneity in resilience operationalization. We extracted data on resilience assessment instruments, theoretical frameworks, and specific measured dimensions (e.g., coping, social support, emotional regulation) to provide a nuanced understanding of resilience conceptualization across studies.

### Databases and search strategy

A comprehensive search was performed across multiple academic databases, including PubMed, Scopus, Web of Science, and Google Scholar, as well as regional databases such as SID, Magiran, and Irandoc. The search strategy was designed to identify relevant publications on resilience among disaster and emergency aid workers from 1989 to November 2023. Keywords were selected based on the research topic, MeSH terms, and expert consultations. Boolean operators (AND, OR) were used to combine keywords related to resilience and disaster relief. The search strategy for each database is detailed in Table 1.

**Table 1:** Search strategy for studies through databases and registers.

Database	Search strategy	No
Scopus	((TITLE-ABS-KEY(resilien*)) AND (TITLE-ABS-KEY(disaster*) OR TITLE-ABS-KEY(catastrophe) OR TITLE-ABS-KEY(crisis) OR TITLE-ABS-KEY(emergenc*)) AND (TITLE-ABS-KEY(“aid worker”) OR TITLE-ABS-KEY(“save worker”) OR TITLE-ABS-KEY(“emergency responder”) OR TITLE-ABS-KEY(“rescue personnel”) OR TITLE-ABS-KEY(“first responder”) OR TITLE-ABS-KEY(rescuer) OR TITLE-ABS-KEY(“rescue worker”) OR TITLE-ABS-KEY(“disaster worker”) OR TITLE-ABS-KEY(“humanitarian aid worker”) OR TITLE-ABS-KEY(worker)))	1734
WoS	((TS = (resilien*)) AND (TS = (disaster*) OR TS = (catastrophe) OR TS = (crisis) OR TS = (emergenc*)) AND (TS = (“aid worker”) OR TS = (“save worker”) OR TS = (“emergency responder”) OR TS = (“rescue personnel”) OR TS = (“first responder”) OR TS = (rescuer) OR TS = (“rescue worker”) OR TS = (“disaster worker”) OR TS = (“humanitarian aid worker”) OR TS = (worker)))	295
Pubmed	(resilien*[TI]) AND (disaster*[TI] OR catastrophe[TI] OR crisis[TI] OR emergenc*[TI]) AND (“aid worker”[TI] OR save worker[TI] OR “emergency responder”[TI] OR “rescue personnel”[TI] OR “first responder”[TI] OR rescuer[TI] OR “rescue worker”[TI] OR “disaster worker”[TI] OR “humanitarian aid worker”[TI] OR worker*[TI])	466
Google Scholar	Resiliency AND “aid workers” AND (disasters or emergencies)	3
Total		3,198

### Inclusion and exclusion criteria

Studies were included if they met the following criteria: (1) investigated resilience in disaster relief workers, (2) were original research studies or theses, (3) provided full-text access, and (4) were published in English or Persian. Exclusion criteria included: (1) lack of full-text access, (2) systematic reviews, meta-analyses, case reports, letters to editors, and conference papers, and (3) pre-printed studies.

### Data extraction and analysis

Data extraction was performed using a standardized form, capturing information on study design, sample characteristics, key findings, and resilience components. Inductive content analysis was employed to identify and categorize the components of resilience. The analysis involved coding the data, identifying themes, and synthesizing the findings into two main groups: resilience traits and resilience processes.

### Study selection process

**Figure 1:**
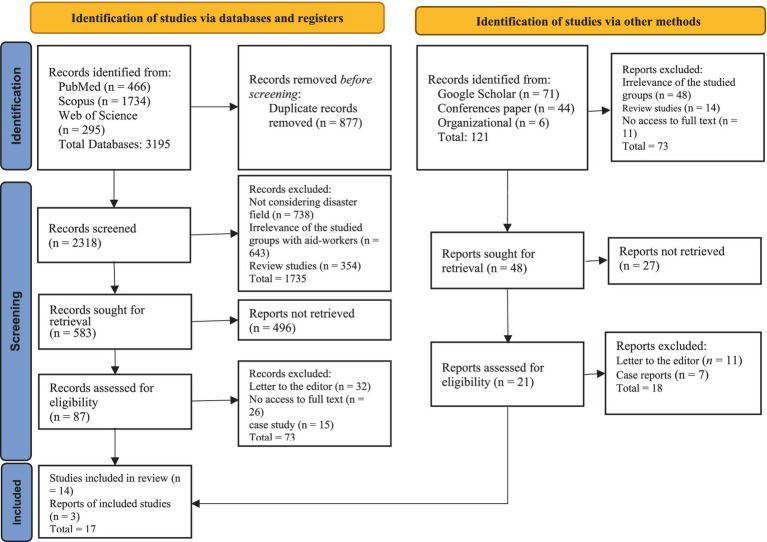
Flow diagram of the selection of studies based on PRISMA 2020.

The initial search yielded 3,198 articles. After removing 877 duplicate records, the titles and abstracts of 2,318 articles were screened for relevance. Articles that did not focus on disaster settings or aid workers were excluded. A total of 1,735 articles were excluded during this stage. The full texts of 583 articles were sought for retrieval, of which 496 were unavailable. To mitigate potential bias from these omissions, related articles and cited references from included studies were examined. Ultimately, 87 articles were assessed for eligibility, and 17 studies met the inclusion criteria and were included in the final analysis (see Figure 1 for the PRISMA flow diagram).

### Quality assessment

The methodological quality of the included studies was assessed using the Mixed Methods Appraisal Tool (MMAT), a validated instrument for appraising qualitative, quantitative, and mixed-methods studies (Hong et al., 2018). The MMAT evaluates studies based on clear research questions, appropriate methodology, and the adequacy of data collection and analysis. Each study was independently assessed by two reviewers, and disagreements were resolved through discussion. The quality scores for the included studies are presented in Table 2.

**Table 2:** Assessment of the quality of the methodology according to the MMAT.

Appraisal of the methodological quality of the included studies																				
No	Author’(s)	Category of study designs	Screening questions (for all types)		Qualitative studies				Quantitative studies					Mixed-methods						Quality score (%)
			S1	S2	1.1	1.2	1.3	1.4	1.5	2.1	2.2	3.3	4.4	5.5	3.1	3.2	3.3	3.4	3.5	
1	Sheikhrabori et al. (2022)	Qualitative	▄	▄				▄												90
2	Scuri et al. (2019)	Observational	▄	▄									▄							80
3	Pink et al. (2021)	Cross sectional	▄	▄										▄						100
4	O’Neil and Kruger (2022)	Mix method	▄															▄		80
5	Nuttman-Shwartz (2014)	Qualitative	▄	▄				▄												80
6	Nishi et al. (2016)	Quantitative	▄	▄									▄							80
7	Mao et al. (2019)	Qualitative	▄	▄					▄											100
8	Mahaffey et al. (2021)	Clinical trial	▄	▄								▄								70
9	Lin et al. (2020)	Cross-sectional	▄										▄							80
10	Katzman et al. (2021)	Interventional	▄	▄								▄								70
11	Gritty (2015)	Qualitative	▄	▄					▄											100
12	Ghodsi et al. (2019)	Qualitative	▄	▄				▄												90
13	Coulombe et al. (2020)	Cross-sectional	▄	▄									▄							90
14	Comoretto et al. (2015)	Mixed-method	▄	▄															▄	100
15	Turner et al. (2021)	Longitudinal study	▄	▄									▄							80
16	Nam (2023)	Mixed- method	▄	▄															▄	90

Methodological quality criteria by type of study include:

S1. Are there clear research questions??

S2. Do the collected data allow to address the research questions??

1.1. Is the qualitative approach appropriate to answer the research question??

1.2. Are the qualitative data collection methods adequate to address the research question??

1.3. Are the findings adequately derived from the data??

1.4. Is the interpretation of results sufficiently substantiated by data??

1.5. Is there coherence between qualitative data sources, collection, analysis and interpretation??

2.1. Is the sampling strategy relevant to address the research question??

2.2. Is the sample representative of the target population??

2.3. Are the measurements appropriate??

2.4. Is the risk of nonresponse bias low??

2.5. Is the statistical analysis appropriate to answer the research question??

3.1. Is there an adequate rationale for using a mixed methods design to address the research question??

3.2. Are the different components of the study effectively integrated to answer the research question??

3.3. Are the outputs of the integration of qualitative and quantitative components adequately interpreted??

3.4. Are divergences and inconsistencies between quantitative and qualitative results adequately addressed??

3.5. Do the different components of the study adhere to the quality criteria of each tradition of the methods involved??

All 17 studies included in this review demonstrated clear research questions and employed methodologies appropriate to address them, as confirmed by the quality assessment via the MMAT. This methodological rigor, coupled with the studies’ relevance to understanding resilience components in disaster aid workers, justified their inclusion in the final analysis.

### Inductive content analysis process

The initial search yielded 3,198 articles. Two reviewers independently screened titles and abstracts after removing duplicates and applying the inclusion/exclusion criteria. Although full-text versions of 496 articles were unavailable, several strategies were employed to minimize potential bias resulting from their exclusion. First, the titles and abstracts of these 496 articles were carefully re-examined to ascertain whether exclusion would likely alter the study’s findings; some were deemed ineligible based on title and abstract information alone, indicating they fell outside the scope of the review. Second, the reference lists of included studies and relevant review articles were scrutinized (“snowballing”) to identify any key publications that might have been missed during the initial database searches. Finally, for a subset of unobtainable articles identified as potentially critical, attempts were made to contact the authors directly to request copies. The research team resolved disagreements through discussion and consensus. The full texts of the remaining promising articles were assessed for methodological quality using the Mixed Methods Appraisal Tool (MMAT), resulting in a final selection of 17 studies for analysis (see Figure 1 for the PRISMA flow diagram).

## Results

A total of 17 studies were included in the final analysis: 9 quantitative (52.94%), 5 qualitative (29.41%), and 3 mixed methods (17.65%). These studies spanned various countries and were conducted between 2015 and 2023. A summary of the included studies is presented in Table 3.

**Table 3:** Characteristics of the ‘included studies’ of resilience among disaster aid workers.

No	DOI	Title of study	First author	Year of issue	Country	Method of study	Key points
1	10.4103/2221-6189.336576	Influential factors of healthcare provider resilience in disasters: A thematic analysis	Sheikhrabori	2022	Iran	Qualitative	Enhancing resilience can be achieved through several approaches, including reducing uncertainty, facilitating access to physical, economic, and human resources, strengthening motivation, and providing comprehensive support systems
2	10.15167%2F2421-4248%2Fjpmh2019.60.1.1134	Training to improve resilience and coping to monitor PTSD in rescue workers	Scuri	2019	Italy	Observational	Several factors play a significant role in building resilience, including education, intervention duration, community support, and the sharing of experiences within the organization and with family and friends.
3	10.1111/joop.12364	Psychological Distress and Resilience in First Responders and Health Care Workers During the COVID-19 Pandemic	Pink	2021	Wales	Cross sectional	It has been shown that resilience is a protective factor against mental distress, anxiety, and depression in frontline responders.
4	10.4102/JAMBA.V14I1.1312	Mindset as a resilience resource and perceived wellness of first responders in a South African context	O’Neil	2022	South Africa	Mix method	Several factors influence the resilience of first responders, including mindset as a source of strength, internal resources, lifestyle choices, and access to external resources.
5	10.1177/15248380145572	Shared Resilience in a Traumatic Reality: A New Concept for Trauma Workers Exposed Personally and Professionally to Collective Disaster	Nuttman-Shwartz	2015	Israel	Qualitative	Resilience is built upon several key components, including education and awareness, alongside individual, family, and organizational factors.
6	10.1539/joh.16-0002-OA	Resilience, posttraumatic growth, and work engagement among health care professionals after the Great East Japan Earthquake: A 4-year prospective follow-up study	Nishi	2016	Japan	Analyctal	The study found a positive correlation between early-career resilience and three characteristics: vigor, dedication, and absorption. This suggests that higher resilience is linked to greater work engagement in rescue activities.
7	10.1016/j.ijdrr.2019.101112	What it takes to be resilient: The views of disaster healthcare rescuers	Mao	2019	China	Qualitative	Study findings attributed resilience in rescuers to personality strengths, coping strategies, social support, and adequate preparations. Additionally, rescuers reported positive life changes following deployment.
8	10.1007/s00420-020-01552-3	The disaster worker resiliency training program: a randomized clinical trial	Mahaffey	2021	USA	Clinical trial	Higher resilience is associated with several positive behaviors, including engaging in healthy practices, managing stress effectively, maintaining spiritual well-being, and utilizing appropriate coping strategies.
9	10.1186/s12888-020-02821-8	Factors associated with resilience among nonlocal medical workers sent to Wuhan, China during the COVID-19 outbreak	Lin	2020	China	Cross-sectional	Factors such as active coping styles, depression, anxiety and training/support provided significantly affected resilience.
10	10.3390/ijerph18094900	First Responder Resiliency ECHO: Innovative Telementoring during the COVID-19 Pandemic	Katzman	2019	USA	Interventional	Evidence-based training, stress reduction strategies, self-confidence, skills, social support, compassion and understanding are important factors that promote resilience.
11	10.1080/13552074.2015.1095542	Building aid workers’ resilience: why a gendered approach is needed	Gritti	2015	Europe	Qualitative	Gender, individual skills, age and cultural background were recognized as important components of resilience in rescue workers
12	10.5812/ircmj.80366	The Resiliency of Humanitarian Aid Workers in Disasters: A Qualitative Study in the Iranian Context	Ghodsi	2019	Iran	Qualitative	The main factors that affect the resilience of disaster relief workers were: disaster scene challenges, self-sufficiency, self-care, job burnout, organizational support and support network.
13	10.3389/fpsyg.2020.580702	Risk and Resilience Factors During the COVID-19 Pandemic: A Snapshot of the Experiences of Canadian Workers Early on in the Crisis	Coulombe	2020	Canada	Cross-sectional	The known important resilience elements were: trait resilience, family functioning, social support, social participation and trust in healthcare institutions
14	10.1080/21577323.2015.1093565	Resilience in Humanitarian Aid Workers: Understanding Processes of Development	Comoretto	2015	England	Mixed-method	Resilience elements in aid workers include: dispositional factors, cognitive factors, environmental protective factors and motivations and coping strategies
15	10.1186/s41018-021-00092-w	Self-efficacy and humanitarian aid workers	Turner	2021	Different countries	longitudinal study	Elements of resilience included adaptive participation, spirituality, emotional regulation and cognitive clarity, behavioral regulation, physical fitness, sense of purpose, and life 16 satisfaction.
16		Resilience in Humanitarian Aid Workers: Examining Expatriates vs National Workers	Nam	2017	Different countries	Mixed- method	Resiliency in aidworkers is related to: meaning, psychological flexibility, training programs and critical incident coping self-efficacy
17	10.1504/IJEM.2017.087220	Managing work-related stress in humanitarian fieldwork: aid workers and resilience resources	Schmidt	2017	Different countries	Qualitative	The elements of resilience in this article included personal resources, external resources, and stress mitigation techniques.

The symbol “▄” indicates that the corresponding resilience component was identified and reported in the referenced study.

**Figure 2:**
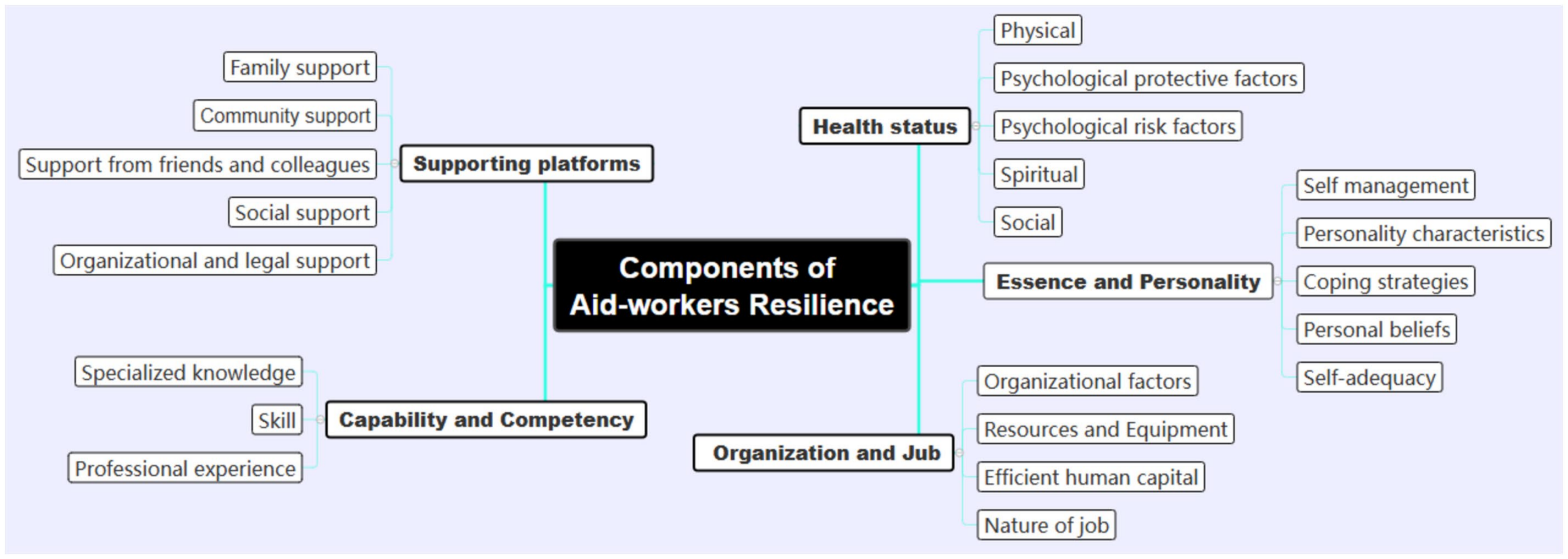
Components of resilience in aid workers in disasters and emergencies.

The final analysis of the 17 studies yielded five key components of resilience in disaster and emergency aid workers, divided into two main groups: traits and processes. Resilience traits include (1) health status, (2) essence and personality, and (3) capability and competency, while the resilience process includes (1) support platforms and (2) organization and job. However, notable differences were observed between studies, particularly in the emphasis placed on specific components. For instance, while some studies highlighted the importance of psychological protective factors (e.g., self-efficacy and stress management) as central to resilience (Brooks et al., 2015; Turner et al., 2021), others emphasized the role of external support systems, such as family and organizational support (Ghodsi et al., 2019; Comoretto et al., 2015). These differences may be attributed to variations in cultural contexts, study populations, and methodological approaches. Table 4: Components identified from the codes extracted from the selected studies (Figure 2).

**Table 4:** Components identified from the codes extracted from the selected studies.

Main categories	Categories	Subcategories	Codes^*^
Resilience trait	Health status	Physical	Physical practices includes adequate sleep, healthy eating and exercise (Buckingham and Brodsky, 2021), physical fitness (Forbes and Fikretoglu, 2018)
		Psychological protective factors	Self-efficacy (Forbes and Fikretoglu, 2018; Brooks et al., 2015), reducing uncertainty (Fletcher and Sarkar, 2013), protective factor against mental distress (Masten, 2001), stress reduction strategies, self-confidence, and compassion (Kirmayer et al., 2009), cognitive factors, environmental protective factors (Mouton, 2022), emotional regulation and cognitive clarity (Forbes and Fikretoglu, 2018), psychological flexibility (Brooks et al., 2015), mindset (focus and attitude) and perceived wellness (Ledesma, 2014), stress management (Buckingham and Brodsky, 2021; Guo et al., 2022)
		Psychological risk factors	A anxiety and depression (Masten, 2001; Kaplan, 2002), Job burnout (DeLuca et al., 2022)
		Spiritual	Spiritual well-being (Buckingham and Brodsky, 2021), spirituality (Forbes and Fikretoglu, 2018)
		Social	Social resilience, community duties, mutual respect (Fletcher and Sarkar, 2013), community support, coordinated assistance (Schreiber et al., 2019), Social cohesion, community resources (Masten, 2001)
	Essence and personality	Self-management	Individual factors (Cutter, 2016), individual skills (Bonanno et al., 2015), Adequate preparations, positive life changes (Bundhoo, 2018), healthy practices, stress management (Buckingham and Brodsky, 2021), self-sufficiency, self-care (DeLuca et al., 2022), emotional regulation, cognitive clarity, behavioral regulation, sense of purpose, life satisfaction (Forbes and Fikretoglu, 2018)
		Personality characteristics	compassion and understanding (Kirmayer et al., 2009), positive life changes (Bundhoo, 2018), vigor, dedication, absorption (Xie and Wong, 2021), personality strengths (Bundhoo, 2018)
		Personal beliefs	Mindset as a source of strength (Ledesma, 2014), factors, which are influenced by personal beliefs including self-confidence and compassion (Kirmayer et al., 2009), sense of purpose (Forbes and Fikretoglu, 2018), motivations (Fletcher and Sarkar, 2013; Mouton, 2022)
		Self-Adequacy	Self-sufficiency (DeLuca et al., 2022), Internal resources (Ledesma, 2014),
		Coping strategies	appropriate coping strategies (Buckingham and Brodsky, 2021), active coping styles (Kaplan, 2002), using coping strategies (Bundhoo, 2018; Mouton, 2022)
	Capability and competency	Specialized knowledge	Education (Schreiber et al., 2019), Evidence-based training (Kirmayer et al., 2009), education and awareness (Cutter, 2016), factors such as training (Kaplan, 2002)
		Skill	significance of skills (Kirmayer et al., 2009), Individual skills (Bonanno et al., 2015)
		Professional experience	Sharing experiences within the organization and with family and friends (Schreiber et al., 2019), transformative experiences (Bundhoo, 2018), age as a form of life experience (Bonanno et al., 2015)
Resilience process	Support platforms	Family support	Sharing of experiences with family (Schreiber et al., 2019), importance of training/support provided, including family support (Kaplan, 2002), Family functioning (Schäfer et al., 2024), familial support systems (Mouton, 2022)
		Social support	Community support (Schreiber et al., 2019), social support and social participation (Bundhoo, 2018; Kirmayer et al., 2009; Schäfer et al., 2024; Mouton, 2022)
		Support from friends and colleagues	Sharing experiences within friends (Schreiber et al., 2019), support of colleagues and managers (Bundhoo, 2018; Kirmayer et al., 2009; Schäfer et al., 2024)
		Organizational and legal support	Providing comprehensive support systems (Fletcher and Sarkar, 2013), sharing of experiences within the organization (Schreiber et al., 2019), organizational support (DeLuca et al., 2022)
	Organization and Job	Organizational factors	Providing comprehensive support systems (Fletcher and Sarkar, 2013), sharing experiences within the organization (Schreiber et al., 2019), organizational factors (Cutter, 2016), organizational support (DeLuca et al., 2022)
		Resources and equipment	Access to external resources (Ledesma, 2014), facilitating access to resources (Fletcher and Sarkar, 2013), adequate preparations including resources and equipment (Bundhoo, 2018), external resources (e.g., necessary equipment and tools) (Guo et al., 2022)
		Efficient human capital	Facilitating access to human resources (Fletcher and Sarkar, 2013), motivated and interested aid workers (Brooks et al., 2015), human factors (Guo et al., 2022)
		Nature of the job	Disaster scene challenges (DeLuca et al., 2022), engagement in rescue activities (Xie and Wong, 2021)

^*^The opposite number of each code corresponds to the study number in Table 3.

## Discussion

While our initial definition characterized resilience as “a dynamic process involving various individual and contextual factors that help maintain and restore psychological well-being after adversity,” we acknowledge the need for greater theoretical precision (Mao et al., 2019; Boldor et al., 2012). Drawing on resilience theory, we refine this definition to: Resilience is a multidimensional construct encompassing not only the capacity to recover from adversity but also to adapt positively, sustain functionality, and experience growth during or after exposure to stressors. It operates across individual, organizational, and systemic levels, mediated by dynamic interactions between intrinsic traits (e.g., self-efficacy, emotional regulation) and extrinsic resources (e.g., social support, institutional policies) (Ledesma, 2014; Masten, 2014; Southwick et al., 2014). This reconceptualization aligns with contemporary frameworks emphasizing resilience as both a process and an outcome. For instance, Ledesma’s synthesis highlights resilience as a “capacity to navigate disruptive challenges while maintaining core organizational or psychological integrity,” which parallels our findings of dual resilience components (traits and processes) (Ledesma, 2014). The identified “support platforms” and “organization/job” factors in our study further reflect the multisystemic nature of resilience theorized by Masten, where environmental scaffolding enables individuals to withstand stressors (Masten, 2014).

Our thematic analysis bridges trait-based and process-oriented perspectives. The “health status” and “capability/competency” components align with trait models emphasizing innate capacities, while “support platforms” and “organizational/job” factors resonate with ecological models prioritizing contextual enablers (Ledesma, 2014; Masten, 2014). This duality mirrors the theoretical integration proposed by Southwick et al., who conceptualize resilience as emerging from gene–environment interactions (Southwick et al., 2014). Notably, our findings extend current theory by identifying “essence and personality” as a distinct trait category specific to aid workers. This aligns with Ledesma’s observation that resilience manifests differently in high-risk professions, where traits like mission-driven purpose buffer against chronic stressors (Ledesma, 2014).

Health status has emerged as a critical component of resilience in disaster aid workers, encompassing physical, psychological (protective and risk factors), social, and spiritual dimensions. Ghodsi et al. (2019) further emphasized the importance of physical fitness for disaster-aid workers’ resilience (Foa et al., 2006). Physical health is a cornerstone of resilience in disaster aid workers, providing a foundation for effective stress management and problem solving during critical incidents (O’Neil and Kruger, 2022). Physical activity enhances physiological resilience through biological changes and improved mental health. Social connections and self-esteem may be impacted by physical activity, but the main mechanisms involve physiological adaptations that improve the stress response and recovery (Mahaffey et al., 2021). Given the centrality of psychological factors to resilience, effective management strategies are essential. Additionally, spiritual beliefs can play a supportive role, as research suggests that religious faith and reliance on greater power can contribute to the mental well-being of aid workers (Ozcan et al., 2021).

Psychological health is another crucial component of resilience in disaster aid workers. Brooks et al. (2015) identified psychological flexibility, stress management skills, and self-awareness as key protective factors (Brooks et al., 2015). Psychological protective factors have been identified as key factors in mental health (Yau and Conwi, 2023). Self-efficacy, highlighted in studies by Turner et al. (2021), Nygaard et al. (2016), contributes significantly to resilience. Turner et al. (2021) reported a positive correlation between higher levels of self-efficacy and resilience (Schmidt, 2017). Conversely, psychological risk factors such as anxiety, depression, and stress, with their associated uncertainty and worry, can negatively impact worker resilience (Lin et al., 2020). Sheikhrabori et al. (2022) further support this notion, demonstrating that factors linked to negative psychological effects in disaster settings diminish resilience (Nam, 2023). Given the central role of psychological factors in resilience, effective management strategies are crucial. On the other hand, expression and management of psychological health may vary across cultural contexts. For example, in collectivist cultures, such as those in East Asia and the Middle East, resilience is often closely tied to family and community support (Ghodsi et al., 2019; Nishi et al., 2016). In individualist cultures, such as those in North America and Europe, resilience may be more closely associated with personal traits like self-efficacy and optimism (Turner et al., 2021; Comoretto et al., 2015). These cultural differences highlight the need for culturally sensitive interventions that consider the unique values and social structures of the target population. For instance, in collectivist cultures, interventions may focus on strengthening family and community networks, whereas in individualist cultures, the emphasis may shift toward personal development and self-care strategies. Spiritual beliefs and religious faith play a significant role in shaping resilience, particularly in diverse cultural contexts. For example, studies conducted in predominantly religious societies, such as Iran and South Africa, have highlighted the importance of spiritual well-being and religious practices in enhancing resilience among aid workers (Ghodsi et al., 2019; O’Neil and Kruger, 2022). In contrast, studies from secular or less religious contexts, such as Europe and North America, have placed greater emphasis on psychological and social support systems (Comoretto et al., 2015; Coulombe et al., 2020). These cross-cultural differences suggest that resilience interventions should be tailored to the cultural and religious backgrounds of the target population. For instance, in religious communities, incorporating spiritual counseling and faith-based support programs may enhance resilience, while in secular contexts, interventions may focus more on psychological training and organizational support.

An analysis of studies focusing on essence and personality revealed that self-management, coping strategies, personal beliefs, self-efficacy, and personality traits are significant components of resilience in aid workers. These findings are consistent with previous research by Brooks et al. (2015), who identified psychological flexibility and stress management skills as key protective factors for resilience (Brooks et al., 2015). Similarly, Mao et al. (2019) found that adaptive coping strategies, such as problem-focused and emotion-focused coping, were associated with higher resilience among disaster healthcare rescuers. Our study builds on these findings by highlighting the importance of context-specific coping strategies, which may vary depending on the type of disaster and the cultural background of the aid workers.

Further research is warranted to explore the differential effects of various coping strategies on disaster aid workers’ resilience across diverse situations. For example, while some studies found that emotion-focused coping strategies effectively reduced psychological distress (Baker and Berenbaum, 2007; Rice et al., 2021), others reported that problem-focused coping strategies were more beneficial in high-stress environments (Mao et al., 2019; Maghan, 2017). These conflicting findings may be due to differences in the types of disasters studied, the duration of exposure, and the cultural backgrounds of participants. For instance, emotion-focused coping may be more effective in acute, short-term disasters, whereas problem-focused coping may be more suitable for prolonged crises. These insights highlight the need for context-specific interventions that consider the unique challenges faced by disaster aid workers in different settings.

On the other hand, personality traits also contribute to resilience in disaster aid workers. These include altruism, hardiness, optimism, and a sense of humor. A study by Mao et al. (2019) highlighted the importance of altruistic motivation. Workers who view helping others and their country as an honor are more likely to participate in relief activities (Mao et al., 2019). Similarly, research suggests that individuals who perceive challenges as opportunities for growth or helping others tend to exhibit greater resilience. This positive outlook likely translates into a stronger desire to aid others and a more resilient approach to challenges (Boldor et al., 2012; Slettmyr et al., 2019). While skills are crucial, hardiness is also seen as a factor that helps aid workers overcome adversity in rescue operations (Mao et al., 2019). Optimism is also a personality trait that plays a prominent role in protecting rescuers from mental disorders. Aid workers who are optimistic experience much lower rates of stress, anxiety, and depression (Yasien et al., 2016). A sense of humor contributes to the resilience of aid workers after they encounter adverse events. This valuable coping mechanism has been recommended for a variety of stressful situations, including catastrophic events (Tanay et al., 2013).

Capabilities and competencies are key components of resilience, as identified in studies that emphasize specialized knowledge, skills, and professional experience. Similarly, various studies have shown that training and preparedness are crucial for assisting workers in facing disasters effectively. Adequate preparation remains essential for aid workers to increase their resilience (Mao et al., 2019). Adequate preparation in essential skills and psychological resilience can equip workers with the competencies necessary to maintain self-efficacy and a sense of control (Mao et al., 2022). Education plays a significant role in preparing individuals with the necessary skills to manage the complexities of exceedingly demanding logistical and emotional circumstances (Scuri et al., 2019). Alexander and Klein (2001) reported that aid workers with proper training experienced fewer negative psychological symptoms after deployment and demonstrated greater resilience (Bonanno et al., 2011). Therefore, resilience requires relevant and specialized training for disaster relief workers. Additionally, they must practice self-care and develop coping mechanisms to empower themselves (Sheikhrabori et al., 2022). Studies have shown that providing information through videos and pamphlets can effectively manage stress (Moghaddam et al., 2021).

Studies have shown that support platforms from family, friends, colleagues, organizations, and the community are important components of resilience. However, the nature and availability of these support systems may vary significantly across cultural contexts. For example, in collectivist cultures, such as those in Asia and Africa, family and community support are often the primary sources of resilience (O’Neil and Kruger, 2022; Nishi et al., 2016). In contrast, in individualist cultures, such as those in North America and Europe, organizational and professional support networks may play a more prominent role (Comoretto et al., 2015; Coulombe et al., 2020). These cross-cultural differences suggest that resilience interventions should be tailored to the specific social and cultural contexts of the target population. For instance, in collectivist cultures, interventions may focus on strengthening family and community ties, while in individualist cultures, interventions may emphasize organizational support and professional development.

Studies have identified organization and job factors as other key components of resilience. These factors include adequate resources and equipment, efficient staffing, and the nature of the job itself. Organizations play a crucial role in building resilience by providing aid workers with the necessary resources, technology, and financial, legal, and psychological support. This comprehensive approach helps improve their psychological well-being (Ghodsi et al., 2019). Comoretto et al. (2015) identified access to resources and equipment as a key environmental factor that strengthens resilience. This is because having the necessary tools empowers rescuers to perform their duties effectively and overcome them effectively (Hull et al., 2002). Human capital is another crucial element of disaster resilience, and organizations play a vital role in this regard by providing training, support, and resources to optimize their workers’ response capabilities (Sheikhrabori et al., 2022). Motivating and sustaining aid workers’ interest and passion is a crucial element of resilience that organizations must prioritize (Ghodsi et al., 2019). We must also consider the inherently stressful and demanding nature of rescue work. Factors such as the chaotic nature of disaster scenes, including overcrowding, inadequate interventions, a lack of security, and burnout, have been reported to significantly impact the resilience of aid workers (Foa et al., 2006).

By identifying the components of resilience in disaster aid workers, this study contributes significantly to a deeper understanding of resilience. These findings can inform the development of targeted interventions to strengthen disaster resilience in aid workers. While this study did not specifically evaluate interventions, previous research has identified several scientifically validated approaches to improving resilience, such as resilience training programs, mindfulness-based stress reduction (MBSR), and cognitive-behavioral therapy (CBT) (Forbes and Fikretoglu, 2018; Mahaffey et al., 2021). Organizations can implement these findings by integrating resilience-building programs into their training curricula, providing access to mental health resources, and fostering supportive work environments. Future research should focus on evaluating the effectiveness of these interventions in real-world settings and exploring how they can be adapted to different cultural and organizational contexts.

This study offers insights for screening and resilience-building in disaster aid workers, emphasizing organizational support and targeted interventions. However, further research is needed to explore contextual and cultural variations in resilience, prioritizing ethical safeguards against retraumatization. The heterogeneity in resilience operationalization across studies poses a challenge for synthesis, highlighting the need for standardized, context-specific measures capturing both trait and process dimensions. Longitudinal studies are also crucial to examine the interplay of individual, organizational, and environmental factors in resilience trajectories.

## Limitations

Several limitations constrain this review. First, while our search was limited to Persian- and English-language databases, this approach may have introduced language bias, potentially excluding relevant research published in other languages. Secondly, the absence of explicit criteria mandating validated resilience instruments could have compromised the consistency and reliability of included findings. Thirdly, the reliance on self-reported data across numerous studies raises concerns regarding potential social desirability and recall biases, as well as subjective interpretations of resilience. Furthermore, the limited extant research on disaster resilience specifically may not adequately reflect the unique stressors inherent in such contexts. An additional limitation is the lack of discussion regarding ethical considerations in studying resilience within disaster settings. This omission is particularly significant given the potential risk of retraumatization when asking aid workers to recall distressing events. Future research should carefully address these ethical concerns and implement appropriate safeguards to protect participants’ psychological well-being.

## Conclusion

This systematic review identified key components of resilience among disaster and emergency aid workers, categorizing them into traits (health status, essence and personality, capability and competency) and processes (support platforms, organization and job) (Alexander and Klein, 2001; Bracken et al., 1995). These findings both confirm and extend current understanding within the broader field of resilience studies.

Specifically, the identification of “essence and personality” as a distinct resilience trait, unique to aid workers, nuances existing models. While frameworks like Ledesma’s (2014) emphasize individual traits and environmental factors, our review underscores the significance of inherent characteristics, such as altruism and a sense of purpose, which may predispose individuals to thrive in high-stress humanitarian settings. This aligns with research suggesting that certain personality profiles are more suited to coping with trauma and adversity (Bonanno et al., 2011). Furthermore, our analysis supports the multi-systemic perspective of resilience (Masten, 2001), highlighting the interplay between individual capabilities and contextual support. The “support platforms” component emphasizes the critical role of social networks and institutional resources in buffering aid workers from burnout and promoting psychological well-being. This resonates with studies demonstrating the protective effects of social support in disaster response (Ozbay et al., 2007).

In terms of humanitarian practice, these findings have significant implications. First, resilience-building programs should adopt a holistic approach, targeting both individual traits and organizational systems. Interventions could focus on cultivating self-awareness, stress management techniques, and promoting a supportive work environment. Second, organizations should prioritize the selection of individuals with inherent resilience traits, while also providing ongoing training and resources to enhance coping skills. Finally, the importance of “organization and job” highlights the need for systemic changes within humanitarian organizations, such as reducing bureaucratic burdens, improving communication, and fostering a culture of psychological safety. By addressing these factors, organizations can create a more resilient workforce capable of effectively responding to the increasing demands of humanitarian crises.

Our study contributes to the field by providing an empirical foundation for targeted interventions and policies aimed at fostering resilience in disaster and emergency aid workers. However, future research should investigate the long-term impact of these interventions, as well as the cultural and contextual factors that shape resilience in different settings.

## Data Availability

The raw data supporting the conclusions of this article will be made available by the authors, without undue reservation.

## Author’s note

Aid workers are often exposed to highly stressful and traumatic situations. This study systematically reviewed the literature on factors that influence resilience among these professionals. The review included both Persian and English-language articles and followed PRISMA guidelines. Key findings highlighted the importance of psychological support, training, and organizational culture in enhancing resilience.
